# piRNA involvement in genome stability and human cancer

**DOI:** 10.1186/s13045-015-0133-5

**Published:** 2015-04-21

**Authors:** Miguel Moyano, Giovanni Stefani

**Affiliations:** Gene Expression Laboratory, Centre for Integrative Biology (CIBio), University of Trento, Trento, 38123 Italy

## Abstract

PIWI-interacting RNAs (piRNAs) are a large family of small, single-stranded, non-coding RNAs present throughout the animal kingdom. They form complexes with several members of the PIWI clade of Argonaute proteins and carry out regulatory functions. Their best established biological role is the inhibition of transposon mobilization, which they enforce both at the transcriptional level, through regulation of heterochromatin formation, and by promoting transcript degradation. In this capacity, piRNAs and PIWI proteins are at the heart of the germline cells’ efforts to preserve genome integrity. Additional regulatory roles of piRNAs and PIWI proteins in gene expression are becoming increasingly apparent.

PIWI proteins and piRNAs are often detected in human cancers deriving from germline cells as well as somatic tissues. Their detection in cancer correlates with poorer clinical outcomes, suggesting that they play a functional role in the biology of cancer. Nonetheless, the currently available information, while highly suggestive, is still not sufficient to entirely discriminate between a ‘passenger’ role for the ectopic expression of piRNAs and PIWI proteins in cancer from a ‘driver’ role in the pathogenesis of these diseases. In this article, we review some of the key available evidence for the role of piRNAs and PIWI in human cancer and discuss ways in which our understanding of their functions may be improved.

## Transposon mobilization and genome instability in cancer

As most cancers stem from the accumulation of mutations, genome instability, defined as a propensity to have mutations, is an ‘enabling characteristic’ of tumor cells [1,2]. There are multiple sources of genome instability in cancer, ranging from exposure to environmental genotoxic substances, to endogenous generation of reactive species of a metabolic origin, resulting in DNA damage. In addition, the human genome carries a plethora of potential insertional mutagens in its own architecture, in the form of transposable elements (TEs) or ‘jumping genes’. While sequences originating from TEs account for a staggering 45% of the entire human genome, only a relatively small set of 80–100 transposable elements are still complete and capable of transposition [3].

The impact of TE mobilization on human cancer has only recently become measurable, thanks largely to progress in the field of high-throughput sequencing technologies. Evolution has endowed cells with a complex arsenal of counter-measures to keep potentially harmful mobilization of TEs in check. One such strategy is based on the action of specialized ribonucleoprotein (RNP) complexes, at the core of which lie members of the PIWI clade of Argonaute proteins and the small, non-coding piRNAs associated with them. While research in recent years has succeeded in unraveling several details of PIWI-interacting RNAs (piRNAs) biogenesis and function in transposon silencing, uncertainties remain on other aspects of their fascinating biology, such as the scope of their function in post-transcriptional regulation of gene expression and their suggested role in human cancer.

This article reviews the intersected fields of piRNA and transposon biology and discusses some of the still rather incomplete evidence in favor of a role of the PIWI/piRNA axis in human cancer.

## Retrotransposons and genome instability

### Transposable elements in the human genome

The existence of transposable elements was first discovered in maize by Barbara McClintock in the 1940s and gained renewed attention about three decades later, when advancement in molecular biology made it possible to appreciate the universality of the phenomenon across living forms. Transposable elements of the human genome can be classified according to their mode of replication as 1) retrotransposons, which are transcribed into an RNA intermediate, and 2) DNA transposons, which do not need transcription to be mobilized (reviewed in [4]).

Much like retroviruses, retrotransposon transcripts need to be retrotranscribed into cDNA by a reverse transcriptase, which is itself encoded by the retrotransposon. In turn, retrotransposons are classified as 1) LTR retrotransposons, legacy of ancient germline retroviral infections and believed to be inactive in humans, 2) long interspersed elements 1 and 2 (LINE-1 and LINE-2 or L1 and L2), and 3) short interspersed elements (SINEs), in turn belonging to the SINE-Alu and SVA classes. While LINEs encode a reverse transcriptase (and are therefore called ‘autonomous’), SINEs do not and depend on the two proteins encoded by LINEs for their own replication and integration. DNA transposons constitute less than 2% of the human genome and depend on various transposases for their mobilization and insertion in their new position in the genome. Only a subset of about 80 to 100 copies of LINE-1 are competent for transposition in humans [5]. These L1s are therefore responsible for the entire retrotransposition activity still present in the human genome, as the two proteins they encode are hijacked by SINEs for their own cycle of retrotranscription and genomic integration.

LINE-1 elements are about 6 kb long and encode two proteins, called ORF1p and ORF2p. The 5′UTR carries an internal promoter, as well as an antisense promoter, whose function is not clear. With their astounding abundance of about 400,000 largely defective copies, they make up close to 17% of the human genome [3]. ORF1p is an RNA-binding protein required for the formation of the retrotransposon particle, while ORF2p carries the two crucial enzymatic activities required for retrotransposition: reverse transcription and endonuclease.

The LINE-1 transposition cycle begins with transcription, driven by the internal promoter, followed by export of the bicistronic mRNA to the cytoplasm, where translation of ORF1p and ORF2p takes place. The ORF1p protein shows a strong preference for binding the transcript molecule that encoded for itself, a phenomenon called *cis*-preference [6]. ORF1p’s biochemical function is not entirely deciphered, but the co-crystal structure of ORF1p and LINE-1 RNA shows that trimers of the protein form a flexible nucleic acid chaperone, around which a single strand of RNA is wrapped, a spatial arrangement likely to contribute to the stability and nuclear import of the complex [7]. An L1 cytoplasmic RNP particle is thus formed, which also includes ORF2p, the second protein encoded by L1 elements.

While the full spectrum of ORF1p binding targets within the transcriptome has not been systematically studied, LINE-1 transcripts are not the only RNA bound by ORF1p: cellular transcripts present in the cytoplasm can be loaded in the complex, albeit at a much lower frequency, leading to the genomic insertion of their corresponding cDNAs in the form of pseudogenes [6]. Furthermore, transcripts from non-autonomous SINE (Alu, SVA) are frequently loaded into these RNP complexes, leading to their insertion into new genomic locations. Finally, the L1 RNP enters the nucleus, where the enzymatic activities of ORF2p lead to genomic integration.

ORF2p is a large protein, with reverse transcriptase and endonuclease activities. The widely accepted biochemical model of L1 integration, called target-primed reverse transcription (TPRT), postulates that, following a nick in one DNA strand corresponding to the recognition sequence (5′-TTAAA-3′), the reverse transcriptase activity of ORF2p extends the DNA 3′ end using L1 RNA as its template [8]. The sequence specificity of the endonuclease is not the sole determinant of the choice of integration site. Recent *in vitro* studies show that the extent of base pairing within the DNA-RNA hybrid formed by the ten bases at the 3′ end of LINE-1 RNA with the nicked DNA affects the efficiency of L1 ORF2p-mediated DNA extension [9]. Such mechanistic studies are beginning to shed light on the complexity of the events leading to integration site choice, which results in a wide range of potential target sites [10].

The biochemical mechanism of L1 integration subsequent to retrotranscription initiation, including synthesis of the second strand of cDNA and ligation of the 3′ ends of the newly retrotranscribed DNA into genomic DNA, is unknown. Such events result in some signature features surrounding LINE-1 integrated in the genome, such as target site duplication (TSD), stretches of identical 5–30 nucleotides at the two ends of the integrated L1 element [11]. The predominant occurrence of 5′ end deletion in integrated LINE-1 elements is most likely a consequence of the 3′ to 5′ direction of reverse transcription and makes the overwhelming majority of them incapable of retrotransposition.

### L1 retrotransposition: when and where

Detection of abundant L1 transcripts and proteins brought forth the concept that transposition takes place actively in germ cells [12,13]. Subsequent studies in transgenic mice, while confirming the presence of L1 transcripts in the germline, failed to reveal frequent genomic insertion in this cell population; L1 elements appear instead to integrate in the genome in the early phases of embryonic development, mostly in somatic tissues, suggesting the presence of post-transcriptional mechanisms specifically devoted to the preservation of genomic integrity from TE insertion in gametes [14].

Despite the presence of multi-layered mechanisms of control in the germline (see below), transposons have obviously managed to trick these mechanisms at various times during evolution, becoming a major driving force in the shaping of genomes, from their prodigious expansion earlier in mammalian evolution to phylogenetically recent differences among primates [15-17]. On the other hand, the presence of L1 transcripts and non-heritable retrotransposition events in somatic cells indicates that TEs contribute to the establishment of mosaicism within an individual [14]. There is abundant evidence of somatic retrotransposition in somatic cell types such as testicular endothelium and Leydig cells, myocardium, and various types of primary human cells in culture [18-21]. Somatic retrotransposition in the brain has been extensively documented, in species ranging from *Drosophila* to human [22] (reviewed in [23]).

In all, the mounting evidence of retrotransposition in somatic tissue, in particular in the brain, challenges the concept that all cells in an individual’s soma have identical genomes (barring the genomic rearrangements in lymphocytes), and supports a scenario where each individual’s soma is a mosaic of somewhat genomically different cell populations. In this perspective, mobilization of TEs can be seen as beneficial, providing a molecular mechanism contributing to the astounding variety of neuron types and connectivity, for instance [23]. On the other hand, the presence of an active insertional mutagen within the genome can also be a powerful source of instability, leading to a variety of human diseases.

### Mechanisms of retrotransposon-induced genome instability

The mechanisms through which TE mobilization can lead to potentially harmful mutations are manifold [4,24]. TEs (mostly Alu elements) provide a vast repertoire of homologous sequences scattered throughout the genome that can be involved in non-allelic homologous recombination (NAHR), which leads to deletions and duplications [25,26]. NAHR-induced mutations are estimated to be vastly more frequent than all the other TE-induced mutations in human cancers (reviewed in [27]). In addition, integration of TEs in exons can cause disease by creating frameshifts, leading to premature stop codons and nonsense-mediated decay, or by inducing exon skipping. Furthermore, TE, in particular Alu, can introduce additional splicing sites upon incorporation into exons (‘exonization’), creating novel, potentially detrimental alternative splicing isoforms [28]. Less frequently, insertions of TEs can also cause large deletions of coding sequence. The relatively weak polyadenylation sites of LINEs are sometimes not processed as such, leading to read-through transcription of sequences belonging to a flanking gene, their incorporation in an active element, and their transduction to a novel genomic location [29].

Additionally, the presence of many polyadenylation sites in the sense strand of LINE-1, while likely to reduce mobilization of functional elements, contributes to worsen the impact of their insertion, by imparting sites of transcriptional pausing and decreasing the overall efficiency of transcription of the host gene [30]. LINE-1 can also disrupt gene expression at the transcriptional level by inducing the antisense transcription of genes flanking its integration site through antisense promoter activity present in its 5′UTR. Additional mechanisms of alteration of gene expression, which might cause human disease, include silencing of flanking genes by heterochromatization of the transposable element as a result of the cell’s control mechanisms and other less frequent molecular phenomena (reviewed in [24]).

The range of human pathologies associated with documented insertion of retrotransposons is vast and expanding. Hancks and Kazazian compiled a list of 96 instances of single-gene disease described in the literature, from hemophilia to diabetes to cancer [4,23,31,32].

### Mobilization of transposons and cancer

As mutagens with very limited sequence requirements for insertion, LINE and SINE are natural candidate pathogenic factors in cancer, a disease caused by mutations. In addition, exposure to some environmental factors that are known to increase cancer risk also promotes TE transcription and promoter demethylation, supporting the notion that transposition might be a causal mechanism of neoplasm [33-35]. Aging, a major risk factor for cancer in humans, is also accompanied by increased TE mobilization in yeast and, more broadly, increased somatic mosaicism in humans [36-38].

Direct evidence of transposon activation in human cancer and transformed cell lines have been gathered over the past 20 years. Components of TEs, both transcripts and proteins, are elevated in cancer compared to normal tissues [33,39-43]. Consistent with a higher level of transposon expression, methylation of transposon promoters is decreased in cancer [44-47] (reviewed in [48]). Both phenomena correlate with worse prognosis and higher levels of metastasis, substantiating a pathogenic role for transposon mobilization [43-47] (reviewed in [48]). These observed correlations grant plausibility to a model in which prolonged exposure to adverse environmental factors in aging organisms leads to increased retrotransposition, with, in turn, increased risk of abrogation of tumor suppressor activities by one of the several mechanisms outlined above.

As it is often the case for cancer mutations, an outstanding question in the field has been whether these transposon activities are ‘driver’ phenomena or consequence of the general derangement of gene expression and genome instability that are hallmarks of cancer. Disruption of genes with a well-established tumor suppressor activity by retrotransposon integration provides a strong correlative evidence for a pathogenetic role for such mutations. A first instance of such occurrence was detected in 1992 in a survey by Southern blot of the APC tumor suppressor gene in 150 colorectal cancer samples. The shift in size of one fragment by several kilobases led to the realization that a LINE-1 sequence was inserted in the gene [49].

Developments in high-throughput sequencing technologies are beginning to allow a full-genome outlook on the impact of TEs on carcinogenesis that was not available just a few years ago. In a recent such study, a survey of 43 full-genome paired-end sequencing databases from five different cancer types revealed 194 instances of novel, cancer-specific somatic insertions [50]. The pool of 64 loci that were affected by these insertions included genes associated with tumor suppressor and cell adhesion functions, with an overall enrichment of frequently mutated genes, a feature associated with a driver role in tumorigenesis [51]. The expression levels of the genes targeted by transposition were decreased, consistent with a functional role of transposon insertion [50].

In disagreement with the conclusions of this report, the analysis of a large dataset of 290 cancer genomes, while identifying a large number (2,850) of new somatic transposition events occurring in cancer, failed to identify insertions with obvious pathogenic implications, such as disruption of tumor suppressor genes, nor did it detect changes in gene expression of the genes targeted by TE integration [52]. Using a method called retrotransposon capture sequencing (RC-seq), 19 hepatocellular carcinoma (HPCC) genomes were analyzed for novel germline and somatic transposition [53]. Two likely L1-mediated tumorigenic mechanisms were unveiled. In 20% of the analyzed genomes, the expression of the tumor suppressor mutated in colorectal cancer (MCC) was drastically reduced by germline L1 insertions, resulting in activation of the oncogenic Wnt/β-catenin pathway. Furthermore, somatic insertion in an intronic transcriptional enhancer resulted in the interruption of a self-repression loop, leading to increased expression of the oncogene ST18 [53].

In summary, while the overwhelming majority of retrotransposon activity in tumors is not likely to be directly driving neoplastic transformation, active retrotransposition is a generator of genomic variability, from which occasionally mutations that impair tumor suppressor genes can arise [52,53].

## Guardians of genome integrity: PIWIs and piRNAs

### PIWI and piRNA biogenesis and mechanism of action

Several layers of control stem mobilization and spreading of transposons in normal cells. Different stages of a retrotransposon’s life cycle are targeted by such control mechanisms: the exonuclease Trex1, which is also involved in cell response to retroviral infection, degrades single-stranded cDNA deriving from retrotransposons, while the APOBEC3 cytidine deaminase family inhibits retrotransposition through a variety of molecular processes [54] (reviewed in [24]). In addition to these mechanisms, studies during the last decade uncovered how the cell leverages a complex level of RNA-based control strategies to inhibit mobile elements at the early stages of transcription as well as post-transcriptionally.

The functional architecture of the PIWI/piRNAs axis shows similarities with other two well-characterized small, non-coding RNA-based regulatory pathways, miRNAs and siRNAs, chiefly among them the role of the RNA component as a specificity factor through base pairing, in the context of a RNP effector complex that includes members of the AGO family of proteins. Biogenesis sets piRNAs apart from the other families of small, non-coding RNAs: while double-stranded or stem-loop precursors of siRNAs and miRNAs are processed by the RNAse III Dicer, piRNAs are mostly transcribed as large (up to 200 kb) single-stranded precursors, which are processed independently from Dicer. In addition, piRNAs form functional complexes exclusively with members of the PIWI clade of Argonaute proteins.

The piRNAs of *Drosophila* and mice can be grouped in three classes according to their origin: 1) repeat-associated piRNAs derived from intergenic loci, called piRNA clusters, that are enriched in transposon fragments in *Drosophila*, zebrafish, and in a subset of piRNA clusters in mice; 2) mRNA-derived piRNAs derived from the 3′UTR of mRNAs; and 3) long, non-coding RNA-derived piRNAs [55]. The biogenesis and functions of repeat-associated piRNAs are much better understood than the other two classes (reviewed in [55,56]).

In the simplest scenario, *Drosophila* gonad somatic cells, the primary transcript is first cleaved, probably by the riboendonuclease Zucchini. The 3′ fragment is incorporated in PIWI proteins and trimmed to a final length of ~25 nt by a 3′ to 5′ exonuclease. The 5′ end residue of the piRNA incorporated in PIWI shows a strong bias for uridine residues, while the 2′ hydroxy group at the 3′ end is methylated by the enzyme Hen1. The final length of piRNAs is likely dictated by the extent of protection from exonuclease trimming, as each PIWI protein binds a subpopulation of piRNAs with slightly different modal sizes (25.7, 24.7, and 24.1 nt for Piwi, Aubergine (Aub), and AGO3, respectively) [57]. The PIWI/piRNA complex enters the nucleus, is targeted to actively transcribed nascent transposon by recognition of sequences complementary to its piRNA component, and inhibits further transcription by recruiting histone methyltransferases, which will lead to the establishment of transcriptionally silent heterochromatin [58].

**Figure 1:**
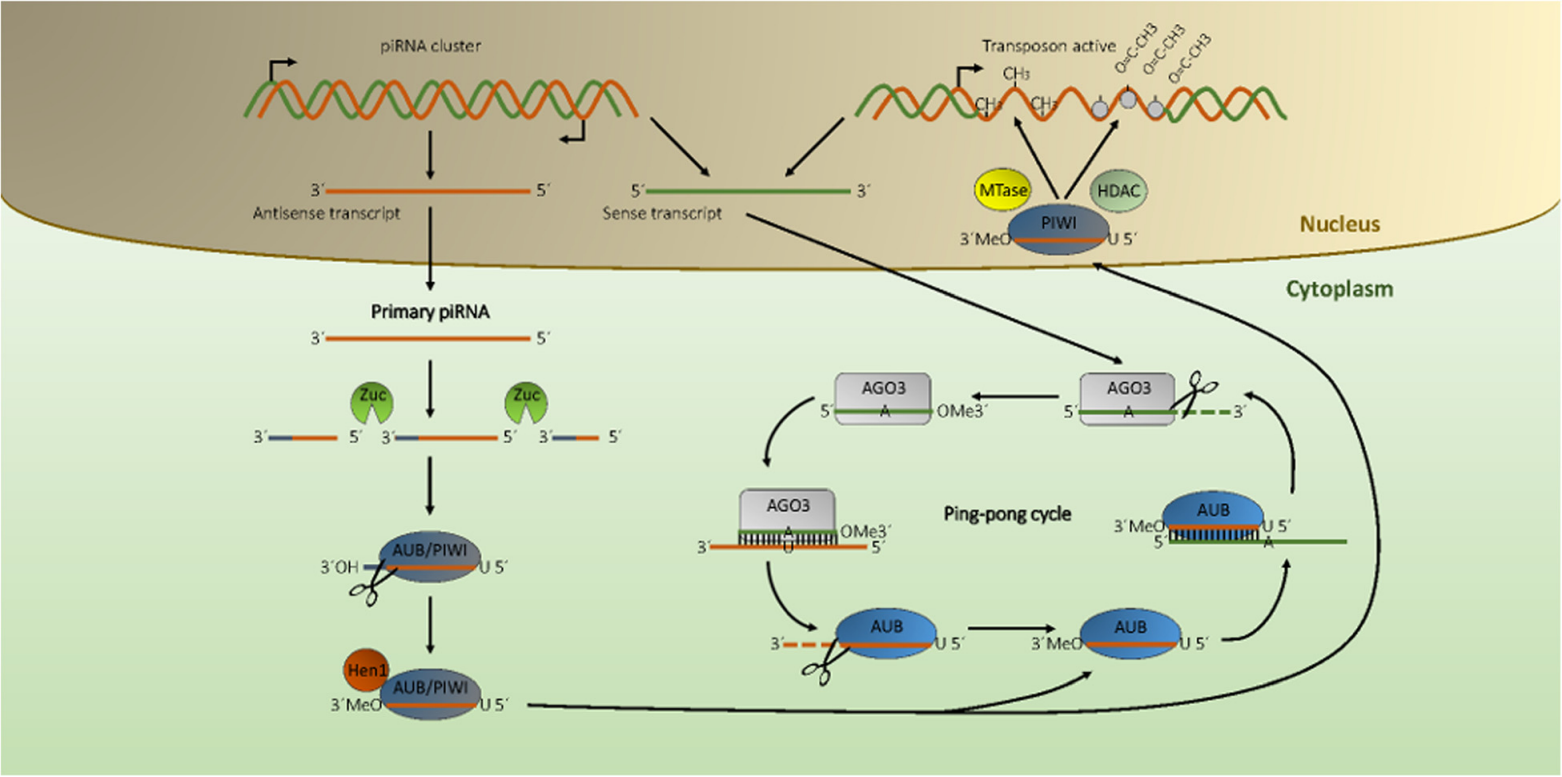
Biogenesis of piRNAs: piRNAs are generated from primary and secondary pathways. While most exhaustively explored in *Drosophila*, the general architecture of these pathways is largely conserved across animals. In the primary pathway, antisense primary piRNAs are cleaved by an endonuclease, most likely Zucchini. Cleaved precursors are incorporated in Aubergine (AUB) or Piwi, with a strong preference for uridine residues at the 5′ end. Subsequently, an exonuclease activity trims the 3′ end to a final length likely to be dictated by protection by the Piwi protein. The 3′ end is then methylated by the Hen enzyme. Aub-bound piRNAs can be amplified in the secondary pathway (ping-pong cycle), which involves the cleavage of a complementary sense transcript by Aub, its trimming, modification, and incorporation in a complex with AGO3. The final outcome is the inhibition of expression of piRNA targets by methylation at the DNA level and by a variety of post-transcriptional mechanisms (not shown in the figure, see text for details).

In *Drosophila* germ cells, this piRNA-mediated inhibition of transposon mobilization is amplified through the ‘ping-pong’ mechanism (Figure 1). Primary piRNAs, antisense to transposons, are incorporated in the Aub PIWI protein and target complementary transposon RNA sequences in the cytoplasm. Aub cleaves transposon RNA between residues complementary to the 10th and 11th piRNA residues. The resulting 3′ fragment of the transposon RNA is incorporated in AGO3, another PIWI protein, trimmed and modified with a 2′ O-methylation, thus forming a secondary piRNA, which will therefore begin with ten residues complementary to the first ten residues of the primary piRNA, with an adenosine as its tenth residue complementary to the first uridine residue of the primary piRNA. When the AGO3-secondary piRNA complex targets antisense strand piRNA cluster transcripts, it will produce new piRNAs identical to primary piRNAs, which will be incorporated in Aubergine complexes, perpetuating the cycle. This mechanism degrades transposon RNA while amplifying the pool of piRNA/PIWI complexes that can inhibit its expression at the transcriptional and post-transcriptional levels (reviewed in [55]).

In mammals, piRNA/PIWI mechanisms display even more variety. The earliest piRNA population, bound to MILI and MIWI2, inhibits transcription of transposons independently of cleavage activity and fades away soon after birth [59]. A mechanism of amplification closely resembling *Drosophila* ping-pong is carried out in prenatal murine testis by the PIWI protein MILI and provides secondary piRNAs bound by MIWI2 [60]. Post-natally, two populations of piRNAs are found in pre-pachytene and pachytene spermatocytes, respectively. The former derives from 3′UTR regions of mRNAs, while the latter, an extremely abundant population of short RNAs, stems from lncRNAs. While genetic ablation experiments show that both these piRNA classes are required for correct spermatogenesis, their molecular function remains a mystery. These very abundant piRNAs do not bear any complementarity to transposable elements, suggesting that they might have a different molecular target. A recent report suggests that pachytene piRNAs and MIWI target up to 40% of mRNAs in elongating spermatids, leading to their deadenylation and degradation during spermiogenesis [61].

Further evidence of the wide scope of the PIWI/piRNA-mediated regulation of gene expression comes from studies in simpler organisms. In *Caenorhabditis elegans*, piRNA-like molecules, called 21U RNAs, are at the center of a complex sequence-scanning system that enables cells to detect non-self sequences such as mobile elements or, occasionally, transgenes and suppresses their expression. This system relies on the production of a large population of secondary siRNAs: upon recognition of a non-self mRNA by 21U RNAs, in a complex with the PIWI protein PRG-1, the so-called 22G RNAs are produced by an RNA-dependent RNA polymerase, using the foreign RNA as template [61-64]. These secondary siRNAs target an effector Argonaute protein, called WAGO-9, to complementary sequences (probably nascent transcripts) in the nucleus, where heterochromatin proteins, DNA methylases, and other factors are recruited to the site of transcription through interactions with WAGO-9, contributing to silencing of the non-self sequences. An opposing scanning system exists, which guarantees the expression of ‘self’ mRNAs. The sequence diversity of 21U RNAs is such that they could conceivably target virtually the entire transcriptome, similarly to mammalian ‘pachytene’ piRNAs: neutralizing their inhibitory effect, yet another group of Argonaute proteins (CSR-1, ALG-3, and ALG-4) forming complex with a separate group of 22G and 26G RNAs promote the proper expression of the genes required for germline development and maintenance [65]. Amazingly, such positive regulators of small RNA/Argonaute complexes are packed in the sperm and transmitted to the zygote, thus providing a vehicle for trans-generational transmission of epigenetic information [65] (reviewed in [66]). Ciliated protozoa also show evidence of genome-scanning mechanisms based on small RNAs that share piRNA features. These scanning RNAs (scnRNAs) regulate programmed DNA elimination, the process by which extensive tracts of DNA are removed from the genome of the haploid micronuclei when they conjugate to form ‘somatic’ macronuclei. In *Tetrahymena* and *Paramecium*, scRNAs mark the DNA sequences that are designated for elimination, while in *Oxytricha trifallax*, piRNAs mark genomic sequences to be retained [67,68].

In addition to chromatin silencing, piRNAs control transposon and mRNA expression in the cytoplasm through degradation and possibly through translational inhibition and intracellular localization (reviewed in [69]). Close to one third of human mRNAs carry transposon-derived sequences in their 3′UTR, making them potential targets for PIWI/piRNA complexes [70]. During maternal *nanos* mRNA elimination in *Drosophila* early development, piRNAs complementary to regions of the 3′UTR and components of the piRNA pathway are required for its deadenylation and degradation [71]. The ability of transposon-derived sequences to induce instability to reporters has also been shown in mammalian systems [72]. These evidence of the ability of the piRNA surveillance system to degrade mRNAs and the pervasive diffusion of transposon sequences in the human genome should be taken into account when considering the possible role of ectopically expressed PIWI proteins and piRNAs in human cancer.

### PIWI, piRNAs, and cancer

PIWI was originally discovered as a factor required for the maintenance of germline stem cells [73]. Members of the PIWI clade Argonaute are expressed in several stem cell populations across organisms and tissues, most robustly in the male germline in adult mammals (reviewed in [74]). Additionally, PIWIs are expressed in a large number of human cancers, of both germline and somatic origin, such as seminomas, multiple myeloma, and prostate, hepatocellular, breast, gastrointestinal, ovarian, and endometrial cancer, among others, as well as murine breast tumors, rhabdomyosarcoma, and medulloblastoma [75-78].

As it is often the case, the question arises of whether such findings point to an active role of PIWI in carcinogenesis, as opposed to representing a mere byproduct of dysregulated gene expression in cancer. Even though the field suffers from a dearth of functional studies, fairly solid correlation between expression of PIWI in cancer and a record of poorer clinical outcome suggest an impact on the biology of tumors. The expression of PIWI proteins in gastric, colorectal, breast, and cervical cancer, soft tissue sarcoma, adenocarcinoma of the pancreas, hepatocellular carcinoma, glioma, and esophageal squamous cell carcinoma correlates with a significant worse prognosis [78-86] (reviewed in [87]).

The mechanisms through which ectopic expression of PIWI affects the clinical outcome of cancers are largely unexplored. In one case, expression of PIWIL2 in three different cell types has been shown to profoundly affect the transcriptome, leading to a marked increase of the antiapoptotic gene Bcl-X_L_, as well as Stat3 and cyclin D1. These changes in gene expression were accompanied by reduced apoptosis, increased proliferation, and transformation [78]. In contrast, the simultaneous ectopic expression of piRNAs with PIWI proteins has been only documented in a handful of cases. In gastric cancer and multiple myeloma cell lines, piRNA-823 levels affect tumor aggressiveness, albeit, strangely, in opposite directions: in gastric cancer cells, piRNA-823 has an overall tumor suppressor activity, while in multiple myeloma, it promotes cancer development, suggesting perhaps functional interactions with cell-specific factors [77,88].

Overall, the current picture of the role of the piRNA/PIWI axis in human cancer is probably incomplete, mirroring the gaps in our grasp of its physiological role. If the sole function of piRNAs was to inhibit the mobilization of transposons, one would expect the ectopic expression of PIWI proteins in cancer to exert an overall antagonistic effect to tumor development and progression. This is not the case in the overwhelming majority of instances in which levels of PIWI have been perturbed in tumor cells: ectopic expression of PIWI actually imparts a more aggressive cancer behavior, and its inhibition reverses such phenotype. One can envision a scenario in which widespread demethylation leads to both activation of transposons and expression of PIWI proteins, or, alternatively, one in which the expression of PIWI proteins is somehow reactive to the activation of transposons through some unknown mechanism [89,90]. Either way, once expressed in the wrong cell types and at the wrong developmental time, PIWI proteins are likely to be engaged not exclusively in the repression of transposon mobilization but also in repressive interactions with RNAs they are not exposed to in germline cells. Post-transcriptional inhibition of gene expression by piRNAs through degradation or translational inhibition could be responsible for the documented tumor-promoting effects. In such a scenario, PIWI/piRNA pro-cancer action would be reminiscent of the oncogenic role of some miRNA-Ago complexes such as miR-155, miR-21, or the miR-17-92 cluster [91-93]. The two modes of action of PIWI/piRNAs, transposon inhibition and post-transcriptional silencing of mRNAs, could still mechanistically be two sides of the same coin, as many mRNAs include extensive transposon-derived sequences [70]. Additionally, it cannot be ruled out at present that PIWI’s and possibly piRNAs’ role in cancer could be completely decoupled from control of transposon mobilization. As mentioned in the previous section, PIWI, piRNAs, and the Argonaute proteins WAGO-9 and CSR-1 and their cognate 22G and 26G RNAs are intertwined in a complex system that allows *C. elegans* to differentiate between ‘self’ from ‘non-self’ RNA [65]. Are mammalian PIWIs and piRNAs also part of an as yet undiscovered mammalian mechanism of vigilance on the proper expression of ‘self’ RNA, and is the derangement of such system somewhat at play in cancer? While vertebrates seem to lack 22G and 26G RNAs, the incredibly high number and sequence diversity of the ‘pachytene’ piRNAs associated to MIWI in mouse provides a repertoire of small, non-coding RNA that could, in principle, target the entire human transcriptome through imperfect complementarity. It is tempting to speculate that the improper activation of such a far-reaching apparatus normally devoted to the monitoring of proper gene expression in the germline may have profound consequence for the biological behavior of cancer.

What are the broader implications of PIWI and possibly piRNA expression in cancer cells? One suggestion comes from a *Drosophila* model of brain cancer: the ablation of the *l(3)mbt* gene, a member of the polycomb group (PcG) of proteins, leads to the development of brain tumors. Expression profiling revealed a ‘*l(3)mbt* tumor signature’ of 102 upregulated genes, which included *piwi*. Mutation of *piwi* was sufficient to suppress *l(3)mbt* malignant growth, providing strong *in vivo* evidence for its requirement for *l(3)mbt* tumor formation. Remarkably, 26 out of 102 upregulated genes that constitute the ‘*l(3)mbt* tumor signature’ are required for germline development and maintenance. They include *vasa* and *aubergine*, which are, like *piwi*, involved in the piRNA pathway and were able to suppress the *l(3)mbt* penotype [94]. An extraordinary coordinated ectopic expression of genes normally restricted to the germline has also been observed in the *C. elegans* ‘soma to germline’ switch upon suppression of *lin*-*35*, an ortholog of RB (retinoblastoma protein), which interacts functionally and associates physically with *l(3)mbt*, as well as in long-lived mutants of the insulin-like pathway [95,96]. Additionally, long-running efforts in the medical community to identify cancer-specific antigen for immunotherapy have resulted in the identification of tens of genes whose expression is exquisitely restricted to the male germline but becomes ectopically activated in a coordinated fashion in a variety of tumors [97]. The products of these genes have been therefore called cancer/testis (CT) antigens. The common thread of the ectopic, coordinated expression of genes that are normally germline-restricted in somatic cancer has led to the speculation that, by conferring biological features typical of the germline, CT antigens might contribute to the biology of cancer cells [97]. Cellular behaviors observed in gametogenesis, such as immortalization, implantation, and migration, may be seen as corresponding to, respectively, transformation, invasion, and metastasis in the context of cancer, while other phenomena such as global hypomethylation, active angiogenesis, and immune evasion are common to both cell types [97]. In this scenario, PIWI proteins ectopically expressed in tumors could contribute to confer features of actively replicating germline stem cells to cancer stem cells.

## Conclusions and perspectives

Seventeen years after the discovery of PIWI and eight after that of piRNAs, the extent of their role in cancer, suggested by ectopic expression and correlation to clinical outcome, is still far from being clearly understood. Addressing the following questions would go a long way in substantially advancing this area of cancer biology:

1. Are piRNAs ectopically expressed in cancer? While PIWI protein expression in cancer is quite extensively documented, piRNAs have been detected in a handful of cases. These include a *Drosophila* model of brain tumor and human multiple myeloma and gastric cancer samples [77,88,94]. The biogenesis of piRNAs is a complex process that needs numerous factors, the expression of many of which has not been studied in most cancers where PIWI is expressed. In the absence of piRNAs, PIWI could perhaps bind other RNAs or engage in functional interactions of a radically different kind.
2. Which genes are regulated by piRNAs and PIWI proteins in cancer? The understanding of the biological functions of any *trans*-regulator of gene expression is greatly advanced by the identification of its functional targets. The field of RNA-binding protein and miRNA-Argonaute biology has benefited from addressing this question through experimental approaches such as high-throughput sequencing of RNA isolated by crosslinking immunoprecipitation (HITS-CLIP) or crosslinking, ligation, and sequencing of hybrids (CLASH) [98,99]. HITS-CLIP for Mili and Miwi in mouse testis reached the unexpected conclusion that Miwi binds mRNAs in the absence of a piRNA guide [100]. Can this finding be confirmed in cancer? Does the identity of genes regulated by ectopically expressed PIWI proteins point to a specific cellular process?
3. Is the ectopic expression of PIWI proteins and piRNAs driving cancer development and progression? More extensive studies in vertebrate animal models are necessary to address this fundamental question. In *Drosophila*, the requirement of *piwi* for brain tumor formation has been elegantly demonstrated in *l(3)mbt*, *piwi* double mutants. Establishing similar models of ectopic expression of PIWI and piRNAs in mammalian model organisms will provide necessary information on their capability to exert a causative role in the initiation and progression of neoplasms.
